# Characterization and evaluation of actinomycete from the *Protaetia brevitarsis* Larva Frass

**DOI:** 10.3389/fmicb.2024.1385734

**Published:** 2024-05-15

**Authors:** Lida Zhang, Tianxin Zhao, Lili Geng, Chao Zhang, Wensheng Xiang, Jie Zhang, Xiangjing Wang, Changlong Shu

**Affiliations:** ^1^Key Laboratory of Agricultural Microbiology of Heilongjiang Province, Northeast Agricultural University, Harbin, China; ^2^State Key Laboratory for Biology of Plant Diseases and Insect Pests, Institute of Plant Protection, Chinese Academy of Agricultural Sciences, Beijing, China

**Keywords:** *Protaetia brevitarsis*, frass, actinomycetes, identification, antifungal, colonization

## Abstract

*Protaetia brevitarsis* larvae (PBL) are soil insects important for the soil organic carbon cycle, and PBL frass not only contains a large amount of humic acid but also affects the diversity, novelty, and potential functions of actinomycetes. Here, we characterized and assessed the actinomycete. The operational taxonomic unit (OTU) data showed that 90% of the actinomycetes cannot be annotated to species, and pure culture and genome analysis showed that 35% of the strains had the potential to be new species, indicating the novelty of PBL frass actinomycetes. Additionally, genome annotation showed that many gene clusters related to antifungal, antibacterial and insecticidal compound synthesis were identified, and confrontation culture confirmed the antifungal activities of the actinomycetes against soil-borne plant pathogenic fungi. The incubation experiment results showed that all isolates were able to thrive on media composed of straw powder and alkaline lignin. These results indicated that PBL hindgut-enriched actinomycetes could survive in soil by using the residual lignocellulose organic matter from plant residues, and the antibiotics produced not only give them a competitive advantage among soil microflora but also have a certain inhibitory effect on plant diseases and pests. This study suggests that the application of PBL frass can not only supplement soil humic acid but also potentially affect the soil microbiota of cultivated land, which is beneficial for the healthy growth of crops.

## 1 Introduction

Soil fauna are important for the soil organic carbon cycle and play an essential role in plant litter decomposition and soil humic substance generation (Morriën et al., 2018). However, soil fauna frass not only contains decomposed organic matter but also contains a large amount of microorganisms (Du B. et al., 2022). Although many researchers have focused on the effect of frass microorganisms on many soil biological processes, such as organic matter decomposition rates (Morriën et al., 2018,), the effects of soil fauna frass microorganisms on soil ecology and their utilization value still need to be further studied.

*Protaetia brevitarsis* is a soil arthropod that has been bred as a resource insect (Hwang et al., 2023). It can feed on decaying plant litter (Wei et al., 2020), and mature larvae contain high-quality protein, fat and a variety of healthy active substances (Lee et al., 2021; Nikkhah et al., 2021). Thus, extensive work has been carried out focused on the larvae, and it has been demonstrated that the digestive tract of *P. brevitarsis* larvae can alter the microbial flora community of organic matter during the digestive process, and lignocellulose decomposing bacteria are enriched in the hindgut (Wang et al., 2022). The altered microbial community in the frass can colonize the decaying organic matter make it more palatable to *P. brevitarsis* larvae (Du S. et al., 2022). Many beneficial microorganisms have also been identified in frass, such as *Bacillus*, *Pseudomonas* and *Streptomyces*, which have been applied as biological insecticides, fungicides, fertilizers and biostimulants (Xuan et al., 2022). Therefore, the microbial flora of frass not only possesses the potential to accelerate organic matter decomposition but also has a positive effect on farmland soil, suppressing plant diseases and promoting plant growth.

Actinomycetes is a group of important microorganisms with ecological function function (Salah El-Din Mohamed and Zaki, 2019; Elshafie and Camele, 2022; Elshafie et al., 2023) and economic value (Chaurasia et al., 2018). In addition to playing major roles in organic matter cycling, actinomycetes were reported to inhibit the growth of plant pathogens in the rhizosphere (Crits-Christoph et al., 2018), degrade high molecular weight compounds in polluted soils (Shahsavari et al., 2019), fix nitrogen in poor soil (Bhatti et al., 2017), improve the availability of nutrients for plants (Li et al., 2020), and be active as plant biostimulants (Kolton et al., 2017). Actinomycetes play an important role in the degradation of organic matter in soil by being able to produce a series of extracellular enzymes that decomposing complex biopolymers like lignocellulose. Lignocellulose is the main structural component of plant biomass, including cellulose, hemicellulose and lignin (Tippelt et al., 2020). Actinomycetes, such as *Acidothermus*, *Nocardia* and *Streptomyces*, play an important role in the degradation of organic matter in soil (Zakalyukina et al., 2021). The capability of actinomycetes to efficiently degrade lignocellulose underscores their vital role in the carbon cycle and their potential utility in biotechnological applications aimed at biomass conversion and sustainable agriculture practices. Furthermore, approximately 70% of reported bioactive secondary metabolites are synthesized by actinomycetes, which have been widely used as insecticides, fungicides/bactericides and antitumor drugs (Vargas Gil et al., 2009) and generate enormous economic value (Salah El-Din Mohamed and Zaki, 2019). In previous work, we detected actinomycetes in *P. brevitarsis* larval frass (Xuan et al., 2022); however, the characterization and evaluation of the *P. brevitarsis* larval frass actinomycete is still insufficient.

In this study, we utilized 16S rRNA sequencing, strain isolation, genome sequencing, and bioassays to assess the diversity, novelty, and potential impacts of frass actinomycetes. This research not only enhances our comprehension of soil fauna effects on soil microbial ecosystems but also unveils a valuable reservoir of actinomycete strain resources.

## 2 Methods

### 2.1 Insects, rearing conditions and sampling

The *P. brevitarsis* population used in this investigation was derived from the wild population in Gongzhuling, Jilin Province, China (Wang et al., 2019). The larvae were raised in a plastic box and kept in an incubator with a relative humidity of 70%, a temperature of 25°C, and a photoperiod of 16 h:8 h. Fully fermented corn straw with approximately 50% moisture content was used to feed the third-instar larvae to produce fresh frass for the subsequent experiment. The corn straw was crushed into approximately 1 cm pieces, and the stocking density was approximately 100 g larvae per 150 g wet corn straw.

To collect the frass, well-grown larvae were collected and cleaned with sterile water, and then the cleaned larvae were kept in an empty clean box at 25°C. The defecated frass was collected after 1 h for further analysis. For hindgut samples, the larvae were immersed in 70% alcohol and washed three times with distilled water. Then, the digestive tract of the larva was dissected, and the hindgut contents were collected. All the samples were preserved at −80°C until further analysis (Xuan et al., 2022).

All requests for additional information and resources should be directed to the lead contact, CS (shuchanglong@caas.cn). This study did not generate new unique reagents.

### 2.2 Actinomycetes isolation from PBL frass

Approximately 1 g of the collected frass was suspended in a 50 mL centrifuge tube filled with 10 mL of sterile water. After shaking at 200 rpm for 30 min, the suspension was serially diluted to concentrations of 10^6^, 10^7^, and 10^8^. Then, dilutions were spread on CPA, DPA, GS, HV and SSA agar plates with 0.5% alkaline lignin. After incubating at 28°C for 7 days, actinomycete-like colonies were picked for further purification and culture. Pure bacterial cultures were stored in 30% (v/v) -glycerol and kept at −80°C for further use (Zhang et al., 2021).

### 2.3 High-throughput sequencing and bioinformatic analyses

Before high-throughput sequencing (HTS), DNA was extracted by a modified protocol using an AxyPrep Multisource Genomic DNA Miniprep Kit. First, approximately 100 mg of frass, hindgut contents or approximately 50 mg of collected cells was suspended and lysed in 600 μL of 4 M guanidine isothiocyanate solution for 2 min. After centrifugation for 5 min at 12,000 × g, 500 μL of supernatant was transferred to the adsorption column. The remaining steps were carried out according to the manufacturer’s protocol, and the DNA yield was used for subsequent analysis.

The 16S rRNA sequencing-based bacterial community analysis was carried out by the method of Du B. et al. (2022). For genome sequencing, 300 bp insert size paired-end libraries for these isolates were constructed by TruSeq DNA PCR-Free Library Prep Kit (Illumina, CA, United States), and after sequencing, the clean reads were produced by removing the low-quality reads (reads with Ns, >20% low-quality bases, or >10 bp that overlapped with adapter sequences). Megahit was employed to assemble the genomes, and the NCBI Prokaryotic Genome Annotation Pipeline (PGAP) was used to annotate the genomes.

Whole-genome-based phylogenetic analysis of these isolates was performed using a CV approach with the CVTree4 web server (Zhao and Hao, 2009). The secondary metabolite biosynthesis gene clusters from these genomes were annotated and identified using the antiSMASH web server (Zhao et al., 2023).

### 2.4 Genomic characterization, average nucleotide identity and DNA–DNA hybridization

Genome sequencing and assembly were performed according to the method described in section 2.3. The EzBioCloud server (Yoon et al., 2017) was used to calculate 16S rRNA gene sequence similarities between strains based on pairwise alignment. ANI and dDDH values were determined between the genomes of the isolates and their closely related species using the ortho-ANIu algorithm from the EzBioCloud server and the genome-to-genome distance calculator (GGDC 2.0) at http://ggdc.dsmz.de. If the dDDH values of strains were lower than the threshold of 70% recommended (Wayne et al., 1987) they represented potential novel species. ANI values below the threshold of 95–96% were also used to delineate new prokaryotic species.

### 2.5 Broad-spectrum resistance of actinomycetes to common pathogenic fungi

To assess the fungistasis potential of the isolates, we used four kinds of soil-borne plant pathogens (*Fusarium oxysporum*, *Pyricularia grisea*, *Rhizoctonia solani* Kühn and *Stagonosporopsis cucurbitacearum*) for plate confrontation tests carried out on PDA medium. The actinomycete spores were incubated in the two kinds of media 30 mm away from the center of the 90 mm medium, while the four kinds of common pathogenic fungi were inoculated into the opposite position of the medium. The pathogenic fungi were inoculated into the medium without actinomycetes as a control. Then, the plates were cultured in an incubator at 37°C. When the pathogenic fungi of the control group grew to the edge of the petri dish, the observed inhibition zone was used as an indicator of the antifungal activity of actinomycetes spores (Liu et al., 2019).

### 2.6 Growth capacity of strains on alkaline lignin medium

As a model compound with a structure similar to that of lignocellulose, alkaline lignin is often used as a raw material for lignin degradation studies. This study explored alkali lignin to investigate the colonization of plant residues in cultivated soil and alkali lignin utilization by strains. The actinomycetes isolated from PBL were inoculated on alkaline lignin medium (0.5% alkaline lignin, 0.2% (NH_4_)_2_SO_4_, 0.1% K_2_HPO_4_, 0.02% MgSO_4_·7H_2_O, 0.01% FeSO_4_·7H_2_O) at 28°C for 7 days, and the growth of actinomycetes on the plate was observed.

### 2.7 Growth capacity of strains on straw powder medium

To investigate potential actinomycete colonization in soil and plant residues, the growth of actinomycetes on straw powder culture medium was explored. The corn straw for feeding PBL was air-dried and crushed with a pulverizer. The actinomycetes isolated from PBL were inoculated on straw powder medium (2.0% straw powder, 2.0% agar). After incubation at 121°C for 20 min, the mixed medium was poured into a 9 mm petri dish and incubated at 28°C for 7 days, and the growth of actinomycetes on the plate was observed.

## 3 Results

### 3.1 Microbial community analysis

**Figure 1:**
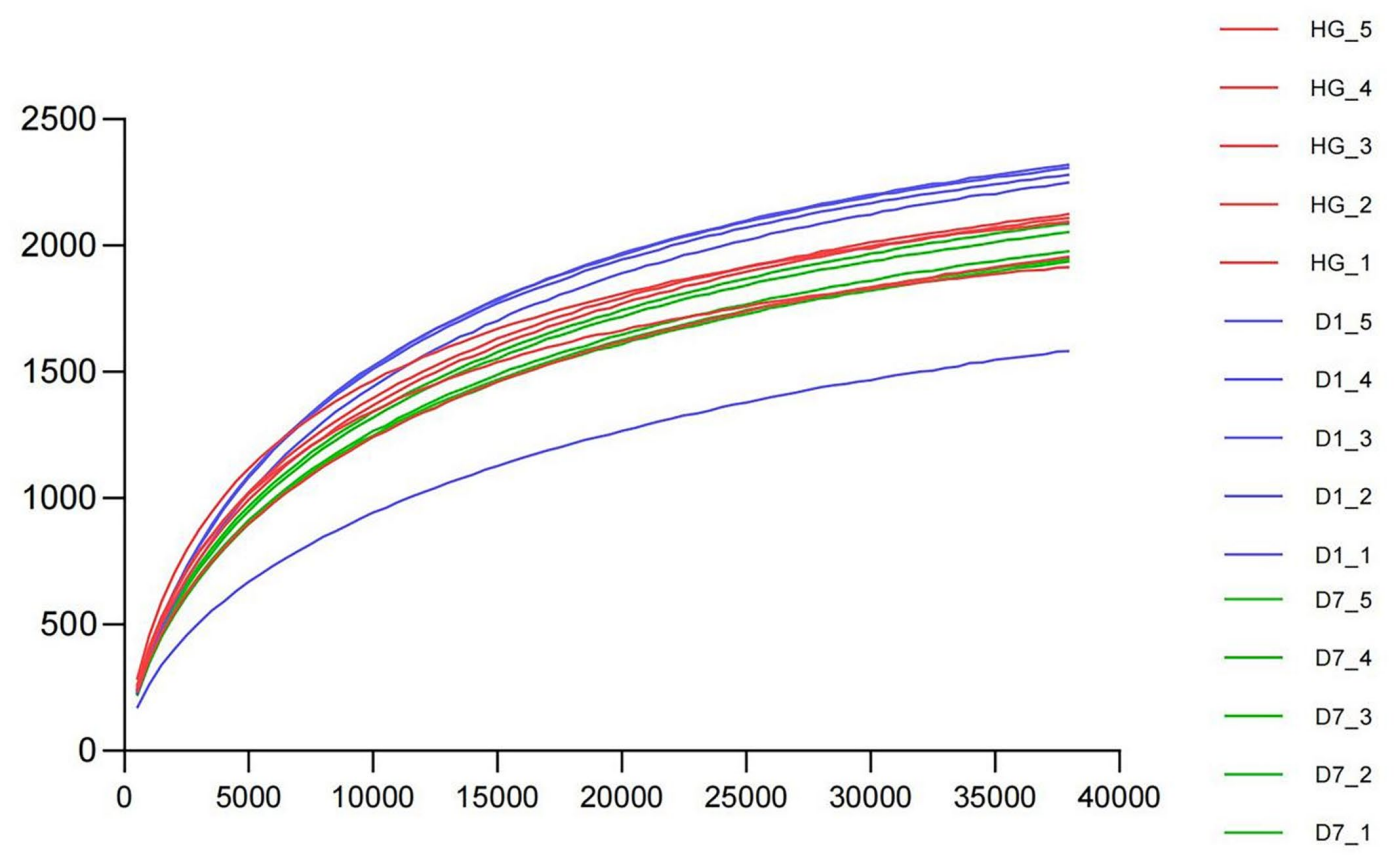
The rarefaction curve applied to OTUs of samples HG, D1, and D7.

We investigated the microbial community structure of PBL hindgut (HG), PBL frass left for one day (D1) and PBL frass left for seven days (D7) by sequencing analysis of the 16S rRNA gene. After trimming the low-quality regions and removing the short reads and chimeras, a total of 851,180 effective tags were identified (Table 1). The rarefaction curve applied to operational taxonomic units (OTUs) showed that all samples reached a plateau, suggesting that all samples were sufficiently sequenced to represent their microbial community diversity (Figure 1). Then, α diversities were analyzed (Table 2), and the OTU and Chao1 of the D1 sample were significantly higher than those of the D7 sample, which represented a significant difference in the sample richness. The result indicating that the frass sample left for one day was richer in microbial species than that left for seven days suggested that environmental changes affect the microbial community structure, and further strain isolation requires selecting samples with the highest abundance of target species. Mean values were contrasted using Duncan’s multiple range test at the 5% (*p* < 0.05) significance level.

**Table 1:** Summary of samples’ sequencing data statistics.

#Label	Trimmomatic	Merge pairs	Primer_match	Zotu
D1_1	77,996	73,251	72,705	64,360
D1_2	68,692	63,607	61,053	56,280
D1_3	75,090	71,412	71,092	58,466
D1_4	79,822	75,780	75,257	65,105
D1_5	86,361	82,494	82,159	70,307
D7_1	55,738	51,415	51,118	38,953
D7_2	70,735	65,524	65,115	46,611
D7_3	68,254	62,155	61,692	47,297
D7_4	73,200	67,922	67,485	53,252
D7_5	74,787	69,098	68,696	53,227
HG_1	79,885	74,942	73,187	66,320
HG_2	82,642	76,949	76,444	58,037
HG_3	85,334	78,779	78,299	63,140
HG_4	78,440	71,505	70,908	55,516
HG_5	76,057	70,701	69,766	54,309

**Table 2:** Summary of samples’ OTU and alpha diversity index.

Sample	OTU	Chaol	Simpson
HG	2956.4 ± 195.46ab	3585.40 ± 202.11ab	0.013 ± 0.002a
D1	3,482 ± 713.36b	4060.72 ± 731.34b	0.026 ± 0.013b
D7	2577.8 ± 133.62a	3153.18 ± 94.10a	0.027 ± 0.006b

**Figure 2:**
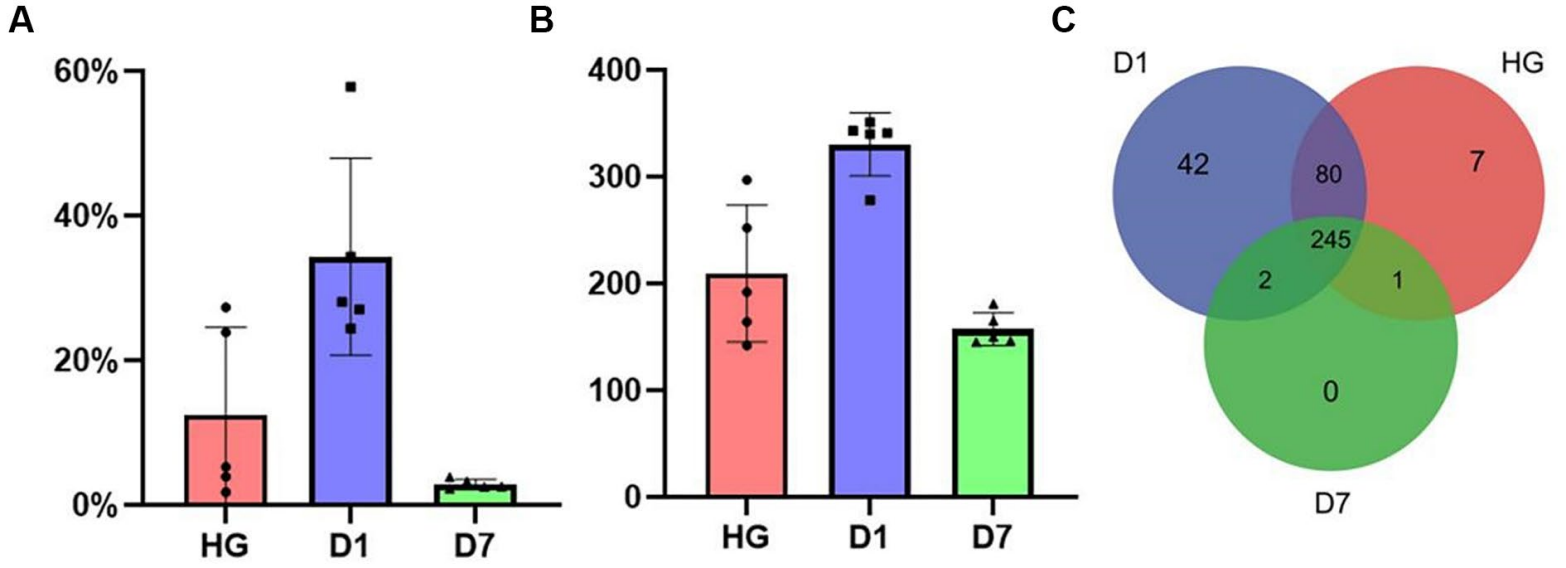
**(A)** The variation in abundance of the phylum *Actinobacteria* in the HG, D1, and D7 samples over with time. **(B)** The variation in OTU abundance of the phylum *Actinobacteria* in the HG, D1, and D7 samples over time. **(C)** A three-way Venn diagram of the number of *Actinobacteria* OTUs of samples HG, D1, and D7.

After OTU annotation, we found that actinomycetes were abundant in these samples. The D1 sample contained 331 *Actinobacteria* OTUs, and the *Actinobacteria* relative abundance reached 34%; it was the most abundant sample of actinomycetes, followed by the HG sample, accounting for 12.4% abundance and 209 OTUs. The D7 sample had the lowest abundance of actinomycetes at 2.9% with 157 OTUs (Figures 2A,B).

Furthermore, a three-way Venn diagram was used to illustrate the variation in actinomycete OTUs between the samples. As shown in Figure 2C, 377 OTUs were assigned to *Actinobacteria*, and there were 245 shared OTUs in all samples, accounting for 65% of the total number of OTUs. Sample D1 contained most of the specific OTUs, while sample D7 had no specific OTUs. Sample D7 contained actinomycetes most similar to the core OTUs, and shared OTUs accounted for the highest proportion 99% of OTUs in D7. In addition, the HG and D1 samples were the most similar to each other, with shared OTUs accounting for 98 and 86% of the two samples, respectively.

**Figure 3:**
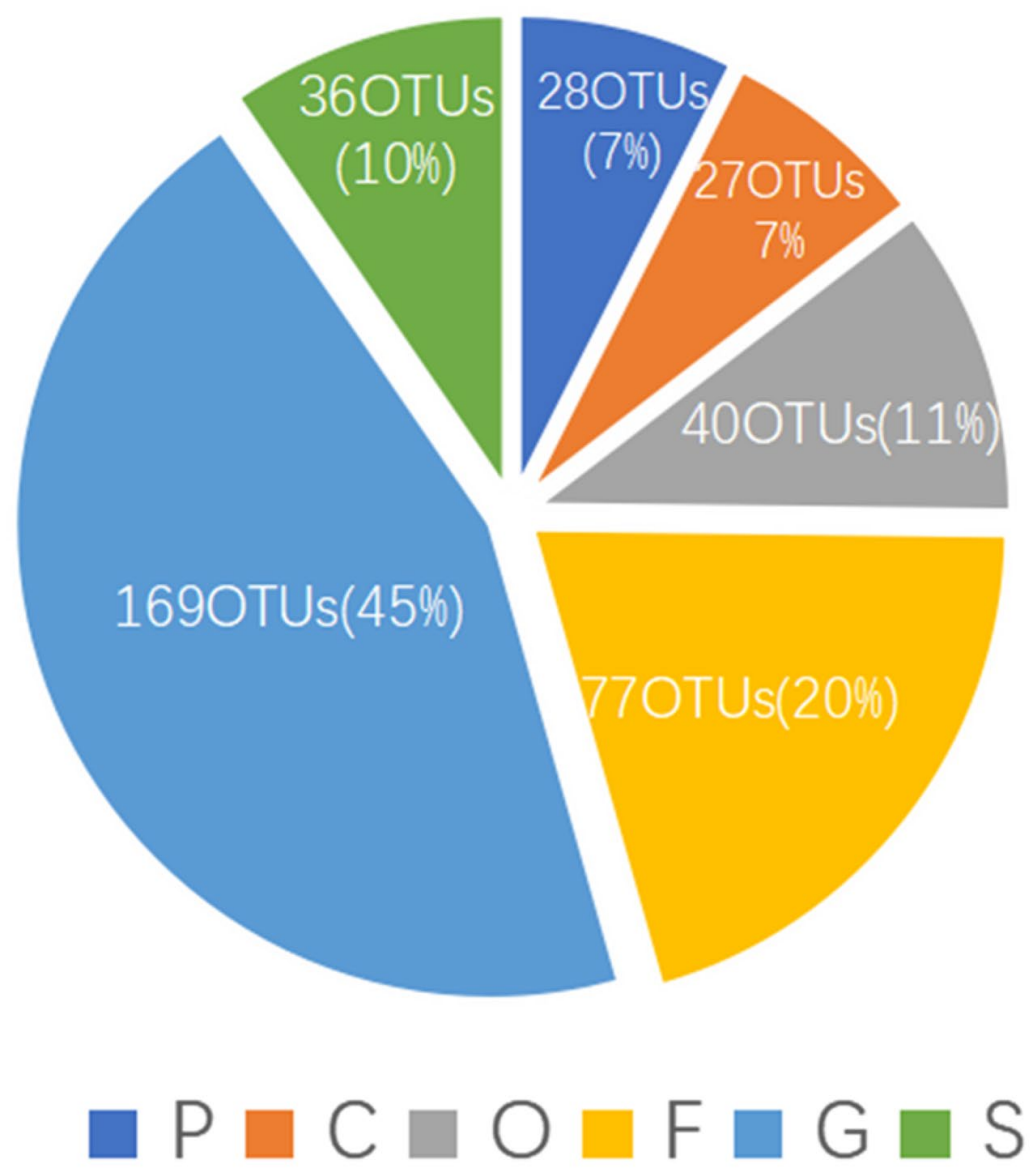
The OTU annotation results in different levels of actinomycetes. P, phylum; C, class; O, order; F, family; G, genus; S, species.

To evaluate the novelty of the actinomycetes contained in the samples, we assessed the OTU annotation data. The data showed that only 36 (10%) OTUs could be assigned to the species level (Figure 3), and the other OTUs may be derived from novel species; they were assigned to the phylum (28 OTUs, 7%), class (27 OTUs, 7%), order (40 OTUs, 11%), family (77 OTUs, 20%) and genus (169 OTUs, 45%) levels. This result indicated that PBL-frass actinomycetes show high novelty.

### 3.2 Actinomycete isolation and identification

**Figure 4:**
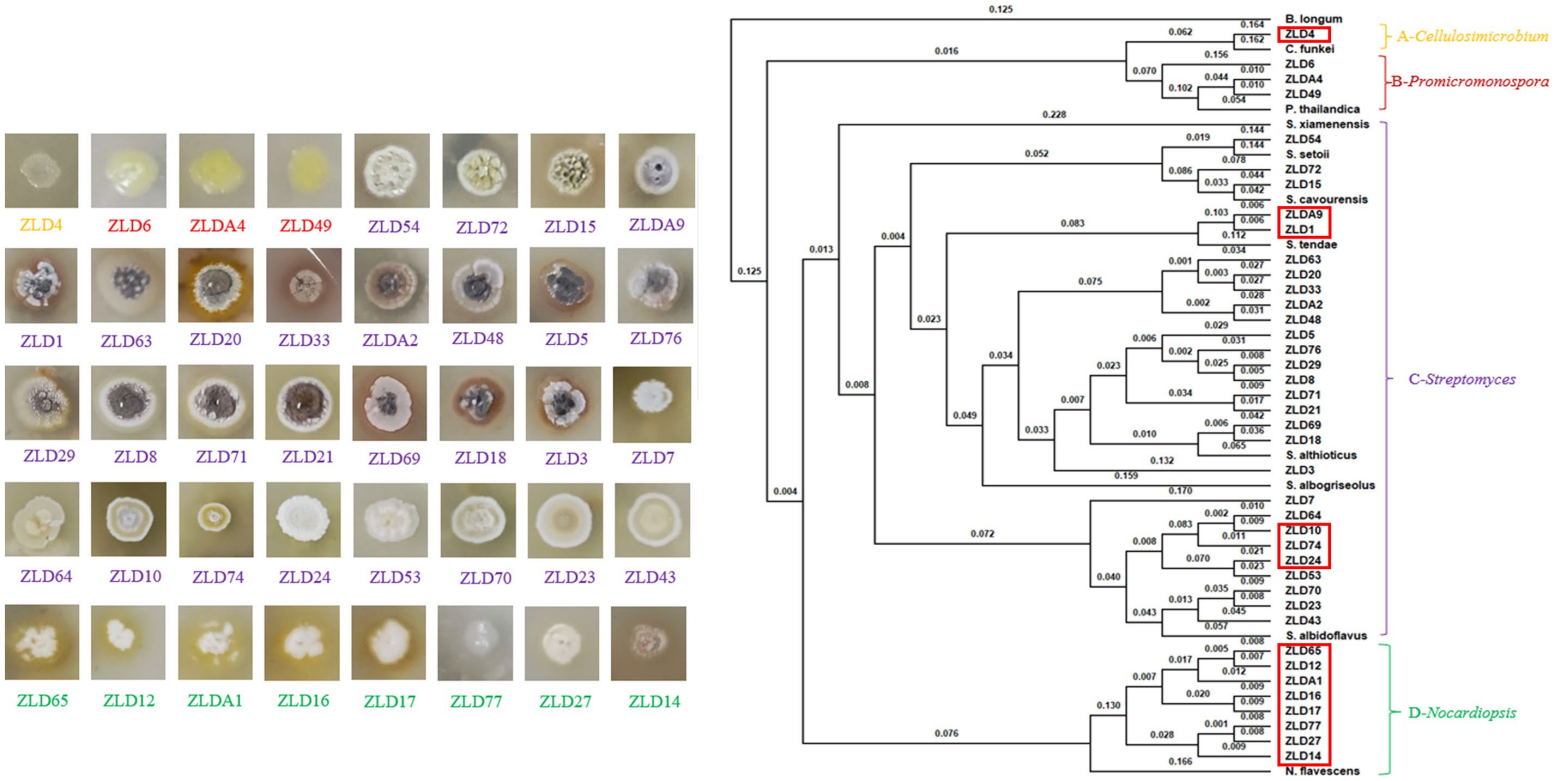
Single colonies of 40 actinomycetes on ISP3 medium and the whole-genome-based phylogenetic tree constructed using the composition vector approach. The distances are shown above the branches.

The actinomycetes were isolated using five different media. After multiple purification passages, 96 actinomycete isolates were isolated from the PBL frass sample. A total of 40 actinomycete isolates with different colony morphological characteristics were eventually obtained (Figure 4).

To better identify these isolates, the draft genomes of these isolates were further obtained by Illumina sequencing. Then, the 16S rRNA gene sequences were extracted and analyzed using the EzBioCloud server for species taxonomy. The results indicated that the 40 isolates contained four clusters, which belonged to the genera *Cellulosimicrobium* (one isolate), *Promicromonospora* (three isolates), *Streptomyces* (28 isolates), and *Nocardiopsis* (eight isolates). Genome-based species taxonomy was performed using a composition vector (CV) approach with the reference genomes of reported strains. According to the phylogenetic relationship, the 40 isolates were assigned to eight species. The isolates in clusters A and B, including one *Cellulosimicrobium funkei* (ZLD4) and three *Promicromonospora thailandica* (ZLD6, ZLD49, and ZLDA4), respectively, presented a coccal shape. Cluster C made up the largest group with 16S rRNA gene sequences identical to *Streptomyces*, including one *Streptomyces setonii* (ZLD54), two *Streptomyces cavourensis* (ZLD72 and ZLD15), two *Streptomyces tendae* (ZLDA9 and ZLD1), 13 *Streptomyces althioticus* (ZLD63, ZLD20, ZLD 33, ZLDA2, ZLD 48, ZLD5, ZLD76, ZLD29, ZLD8, ZLD71, ZLD21, ZLD69, ZLD18, and ZLD3), and nine *Streptomyces albidoflavus* (ZLD7, ZLD64, ZLD10, ZLD74, ZLD24, ZLD53, ZLD70, ZLD23, and ZLD43). Some of these isolates have pale aerial mycelium and do not produce soluble pigment, such as strains ZLD7 and ZLD64; other isolates have deep aerial mycelium and produce no soluble pigment, such as strains ZLDA9 and ZLD76; and some strains have deeply colored aerial mycelium and produce soluble pigment, such as strains ZLD20 and ZLD33. Cluster D, including eight *Nocardiopsis flavescens* (ZLD65, ZLD12, ZLDA1, ZLD16, ZLD17, ZLD77, ZLD27, and ZLD14), make up the second largest group. The isolates in this group only have substrate mycelium, and some of them produce soluble pigments, such as strains ZLD65 and ZLD12. Therefore, morphological and genetic data show that actinomycetes isolated from PBL frass have rich species diversity.

### 3.3 Average nucleotide identity and DNA–DNA hybridization

According to 16S rRNA sequence identity data, some potential novel actinomycetes were isolated in this research. The digital DNA–DNA hybridization (dDDH) values of 14 strains, which accounted for 35% of all actinomycetes, were lower than the threshold of 70% recommended (Wayne et al., 1987) thus, they represented potential novel species. The average nucleotide identity (ANI) values of 13 strains, which accounted for 32.5% of all actinomyces, were below the threshold of 95–96% and were also used to delineate new prokaryotic species. As shown in Table 3, fourteen strains may represent potential novel species, including one *Cellulosimicrobium* (ZLD4), five *Streptomyces* (ZLDA9, ZLD1, ZLD10, ZLD74, ZLD24) and eight *Nocardiopsis* (ZLD65, ZLD12, ZLDA1, ZLD16, ZLD17, ZLD77, ZLD27, and ZLD14). The locations of potential new species are marked in red frames in Figure 4. According to the results of DNA hybridization between strains, all potential new species of actinomycetes can be divided into at least 10 potential new species. The data showed that PBL frass contained many potential novel species, which accounted for 35% of the isolates in this study, suggesting that PBL frass is a valuable resource that contains many novel actinomycete species.

**Table 3:** Summary of the digital DNA–DNA and ANI values in some isolates.

Query strain	Subject strain	dDDH (d4, in %)	C.I. (d4, in %)	OrthoANIu value (%)
ZLD 4	Cellulosimicrobium protaetiae BI34T	35.4	[32.9–37.9]	86.81
ZLD A9	*Streptomyces tendae* JCM 4610	60.0	[57.2–62.8]	94.75
ZLD 1	*Streptomyces tendae* JCM 4610	60.0	[57.2–62.8]	94.87
ZLD 10	*Streptomyces albidoflavus* NRRL B-1271	65.1	[62.2–68.0]	95.97
ZLD 74	*Streptomyces albidoflavus* NRRL B-1271	65.1	[62.1–67.9]	96.01
ZLD 24	*Streptomyces limosus* NBRC 12790	66.2	[63.2–69.0]	95.96
ZLD 65	*Nocardiopsis flavescens* CGMCC 4.5723	36.5	[34.1–39.1]	88.75
ZLD12	*Nocardiopsis flavescens* CGMCC 4.5723	22.8	[20.5–25.2%]	88.71
ZLD A1	*Nocardiopsis flavescens* CGMCC 4.5723	36.6	[34.1–39.1]	88.72
ZLD16	*Nocardiopsis flavescens* CGMCC 4.5723	36.6	[34.2–39.2]	88.75
ZLD17	*Nocardiopsis flavescens* CGMCC 4.5723	36.50%	[34.1–39%]	88.72
ZLD 77	*Nocardiopsis flavescens* CGMCC 4.5723	36.6	[34.1–39.1]	88.82
ZLD 27	*Nocardiopsis flavescens* CGMCC 4.5723	36.5	[34.1–39.0]	88.70
ZLD 14	*Nocardiopsis flavescens* CGMCC 4.5723	36.6	[34.1–39.1]	88.77

### 3.4 Analysis of secondary metabolites

To evaluate the secondary metabolite biosynthesis potential of these isolates, the secondary metabolite biosynthesis gene clusters of 40 isolates were annotated by antiSMASH 6.0. In total, 215 highly consistent clusters (identity >70% with the reference cluster) were identified. The clusters can be divided into type I lasso peptides, cyclic dipeptides, RiPPs, polyketides, terpenes, NRPs and some other clusters. The identity data showed that only 43% of secondary metabolite gene clusters had more than 95% consistency with the reference, which suggested that the gene clusters in these actinomycetes had not only functional diversity but also good novelty.

First, we summarized the most widely distributed gene clusters of these strains. Albaflavenone (present in 20 strains, 50%), as the most widely distributed cluster among strains, ranked second only to geosmin, the odor source of actinomycetes, and belongs to terpenes, which have antibacterial activity. In addition, we noted that alkylresorcinol belongs to the PKS, which has insecticidal properties, and was present in 16 strains (40%). Xenematide, as a cyclic dipeptide, has moderate antibacterial activity and was present in 15 strains (37.5%). Desferrioxamine was applied to the treatment of anemia and acute iron poisoning and was present in 15 strains (37.5%).

Afterward, we evaluated the known functions of these gene clusters. Among them, we found that five kinds of gene clusters have antibacterial activities, with a total of 46 related gene clusters identified and present in 31 strains, accounting for 77.5% of strains in this research; five kinds of gene clusters inhibit fungi, with a total of 17 related gene clusters detected and present in 14 strains, accounting for 35% of strains in this research; and two kinds of gene clusters have insecticidal properties, with a total of 21 related gene clusters detected and present in 21 strains, accounting for 52.5% of strains in this research. In addition, one plant growth-promoting gene cluster was identified.

**Figure 5:**
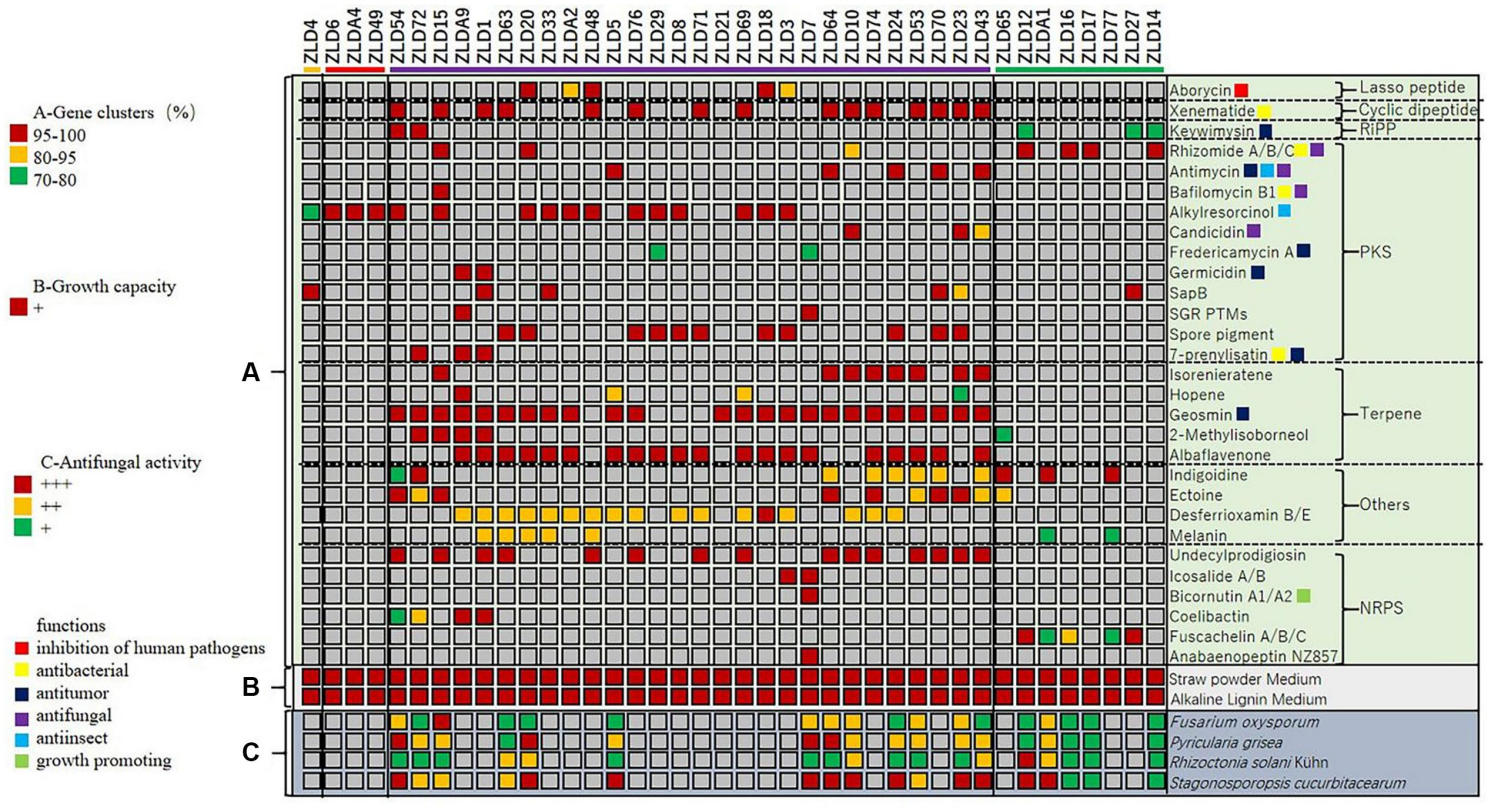
Cluster analysis of secondary metabolite biosynthesis, growth capacity, and antifungal activity of the strains.

In general, strains ZLD15 and ZLD1 contained the most rich clusters (11); of these, ZLD15 contained two kinds of antifungal, three kinds of antibacterial, and one kind of insecticidal clusters, and ZLD1 contained three kinds of antibacterial clusters. We also summarized the strains containing the most functional gene clusters related to antifungal, antibacterial and insecticidal activities. Except for ZLD15, which contained six kinds of functional gene clusters, ZLD43 was the strain with the most functional gene clusters, containing five kinds (two antifungal, two antibacterial, and one insecticidal). The different types of secondary metabolite gene clusters predicted in this study are shown in Figure 5.

### 3.5 Antifungal bioassay

The antifungal effects of the actinomycetes on plant pathogenic fungi were assessed by confrontation culture analysis (Supplementary Figure S1). Fifteen of the isolates showed antifungal activity against four kinds of soil-borne plant diseases. There were 13 *Streptomyces*, including one *S. setonii* (ZLD54), two *S. cavourensis* (ZLD72 and ZLD15), three *S. althioticus* (ZLD63, ZLD20, and ZLD5), and seven *S. albidoflavus* (ZLD7, ZLD64, ZLD10, ZLD24, ZLD53, ZLD23, and ZLD43) and two *Nocardiopsis flavescens* (ZLD14 and ZLDA1). Among them, four potential new species isolates showed antifungal effects (ZLD10, ZLD14, ZLD24, and ZLDA1).

### 3.6 Growth capacity of strains on alkaline lignin and straw powder media

To evaluate the colonization potential of these strains in soil organic matter, we analyzed the growth capacity of these strains on straw powder medium and lignin medium. The results showed that all isolates could grow on straw powder medium and alkaline lignin medium (Supplementary Figure S2).

## 4 Discussion

In the ecosystem, terrestrial arthropods, such as PBL, largely contribute to the decomposition of plant litter and the formation of soil organic matter. In Asia, PBL have been bred as a resource insect to transform agricultural wastes and produce insect protein, and the frass produced in the process of insect breeding has a high content of humic acid (HA), whose structure is consistent with that from cultivated land soil (Li et al., 2019). Therefore, it is an effective strategy to use PBL to transform straw and apply frass as organic fertilizer for cultivated land organic matter supplementation. Moreover, our findings revealed the presence of numerous microorganisms in the frass, which will be introduced into the soil upon application of PBL frass (Xuan et al., 2022). Consequently, it is crucial to assess the influence of these frass microorganisms on the microbial ecological environment of cultivated land.

It has been confirmed that the PBL intestinal tract is rich in microorganisms that can utilize lignocellulose (Wang et al., 2022), and microbial community research has confirmed that these microorganisms contain many agriculturally beneficial microorganisms (Xuan et al., 2022). In this study, we focused on key soil microorganism actinomycetes, which not only play an important role in soil organic matter decomposition and the carbon cycle but can synthesize many active substances that play an important role in soil microbial ecology and have many effects on plants.

The results of microbial community analysis showed that 90% of the actinomycetes could not be assigned to the species level, and pure culture analysis showed that 40 actinomycetes could be divided into at least 4 genera and 27 different species, and 35% of the actinomycetes could be potential new species, which showed that PBL frass contains a large number and a good diversity of actinomycetes. The growth capacity in alkaline lignin and straw medium showed that all isolates could grow on straw powder medium and alkaline lignin medium, which suggested that these actinomycete microorganisms may have the opportunity to colonize plant residues in cultivated soil or use the residual lignocellulose in PBL frass to survive when applied to cultivated land. The antifungal bioassay showed that 15 of the isolates showed broad antifungal activity against four kinds of soil-borne plant diseases. The secondary metabolite biosynthesis potential evaluation showed that some of the secondary metabolite gene clusters have anti-bacterial, anti-fungal and anti-insect functions, which suggests that the actinomycete microorganisms in PBL frass may not only inhibit plant pathogens but also control some plant pest species. Therefore, the actinomycete microorganisms in PBL frass will play an active role in the soil ecosystem when applied to cultivated land.

Because of the important application value of the secondary metabolites synthesized by actinomycetes, people are continuously exploring new actinomycete resources (Ling et al., 2020). In this investigation, the data confirmed that the PBL frass microbial community contains many new actinomycetes species. In particular, the rare actinomycetes *Nocardiopsis*, *Promicromonospora*, and *Cellulosimicrobium,* apart from *Streptomyces*, whose genera are only found in less explored environments, were detected and isolated in this study (Stackebrandt and Prauser, 1991; Karthik et al., 2016; Zhang et al., 2016). Furthermore, these actinomycete strains are predicted to encode many secondary metabolite synthesis gene clusters with the potential to synthesize a variety of active substances, such as bacteriostasis, insect resistance and cancer resistance. In addition to diversity, these gene clusters also have high novelty, and more than half of the detected gene clusters are less than 95% consistent with the reference sequence. Variation at the gene level may impact the synthesis efficiency and even the structure of the final products; thus, the related strains are worthy of further study. In addition, some recent studies have suggested that insect-associated *Streptomyces* inhibit antimicrobial-resistant pathogens more effectively than soil *Streptomyces* (Chevrette et al., 2019). Thus, PBL frass is a valuable new source for actinomycete resource mining.

## 5 Conclusion

In summary, OTU data, and strain isolation and identification results revealed the novelty of actinomycetes. Gene cluster analysis predicted that these strains have antifungal, antibacterial and insecticidal properties. Plate confrontation experiments showed the inhibitory effect of some actinobacteria on soil-borne plant diseases. Culture experiments showed that the strain could colonize lignocellulose. The microbial diversity and isolated actinomycetes from PBL frass were analyzed for the first time in this study. The traditional source of actinomycetes isolation mainly comes from the soil; however, from PBL frass, we showed a large dominance in abundance and novelty of the actinomycete from PBL frass and the potential of the strains to colonize soil organic matter and resist plant diseases and pests. The results of this study illustrated that the PBL frass was rich in actinomycetes, and the PBL frass actinomycetes not only have rich genetic diversity but also have the potential to colonize cultivated soil, inhibit plant pathogens and pests, and may improve the microbial ecology of cultivated soil for crop production. In addition, the novelty in species and secondary metabolite synthesis gene clusters suggests that PBL frass is a valuable actinomycete resource. Therefore, this study not only presents a valuable actinomycete resource but also provides guidance for the further application of PBL frass.

## Data availability statement

The strains whole genome sequence data reported in this paper have been deposited in the Genome Warehouse (2021) in National Genomics Data Center (2024), Beijing Institute of Genomics, Chinese Academy of Sciences / China National Center for Bioinformation, under accession number ZLD4 (GWHESNE00000000); ZLD6 (GWHESNF00000000); ZLDA4 (GWHESOL00000000); ZLD49 (GWHESNH00000000); ZLD54 (GWHESOP00000000); ZLD72 (GWHESNI00000000); ZLD15 (GWHESNJ00000000); ZLDA9 (GWHESNK00000000); ZLD1 (GWHESNL00000000); ZLD63 (GWHESNM00000000); ZLD20 (GWHESNN00000000); ZLD33 (GWHESNO00000000); ZLDA2 (GWHESNP00000000); ZLD48 (GWHESNQ00000000); ZLD5 (GWHESOM00000000); ZLD76 (GWHESNR00000000); ZLD29 (GWHESNS00000000); ZLD8 (GWHESNT00000000); ZLD71 (GWHESNU00000000); ZLD21 (GWHESNV00000000); ZLD69 (GWHESNW00000000); ZLD18 (GWHESNX00000000); ZLD3 (GWHESNY00000000); ZLD7 (GWHESNZ00000000); ZLD64 (GWHESOQ00000000); ZLD10 (GWHESOA00000000); ZLD74 (GWHESOB00000000); ZLD24 (GWHESOC00000000); ZLD53 (GWHESOD00000000); ZLD70 (GWHESOE00000000); ZLD23 (GWHESOF00000000); ZLD43 (GWHESO(G00000000); ZLD65 (GWHESOH00000000); ZLD12 (GWHESOI00000000); ZLDA1 (GWHESOJ00000000); ZLD16 (GWHESOK00000000); ZLD17 (GWHESON00000000); ZLD77 (GWHESOO00000000); ZLD27 (GWHESND00000000); ZLD14 (GWHESNC00000000) that is publicly accessible at https://ngdc.cncb.ac.cn/gwh.

## Ethics statement

The manuscript presents research on animals that do not require ethical approval for their study.

## Author contributions

LZ: Writing – review & editing. TZ: Writing – original draft. LG: Writing – review & editing. CZ: Writing – review & editing. WX: Writing – review & editing. JZ: Writing – review & editing. XW: Writing – review & editing. CS: Writing – review & editing.
