# Supplementary material for: Characterization and evaluation of actinomycete from the Protaetia brevitarsis Larva Frass
Source: Front Microbiol. 2024 May 15;15:1385734. doi: 10.3389/fmicb.2024.1385734 (PMC11133513; doi:10.3389/fmicb.2024.1385734)
Supplement: Supplementary file 1 [file Image_1.pdf]

Characterization and Evaluation of Actinomycete from the *Protaetia brevitarsis* Larva Frass

Lida Zhang[1,2#], Tianxin Zhao[1#], Lili Geng[2], Chao Zhang[1,2], Wensheng Xiang[1,2], Jie Zhang[2], Xiangjing Wang[1,2,*], Changlong Shu [2,*]

[1]Key Laboratory of Agricultural Microbiology of Heilongjiang Province, Northeast Agricultural University, No. 59 Mucai Street, Xiangfang District, Harbin 150030, People's Republic of China

[2]State Key Laboratory for Biology of Plant Diseases and Insect Pests, Institute of Plant Protection, Chinese Academy of Agricultural Sciences, Beijing, People's Republic of China

Tel: +86-451-55190413

Fax: +86-451-55190413

*** Correspondence:**

*Corresponding author: Xiangjing Wang (wangneau2013@163.com) and Changlong Shu (shuchanglong@caas.cn).

#Lida Zhang and Tianxin Zhao contributed equally to this work.

Contents:

Supplementary Figure 1

Supplementary Figure 2

Figure S1.

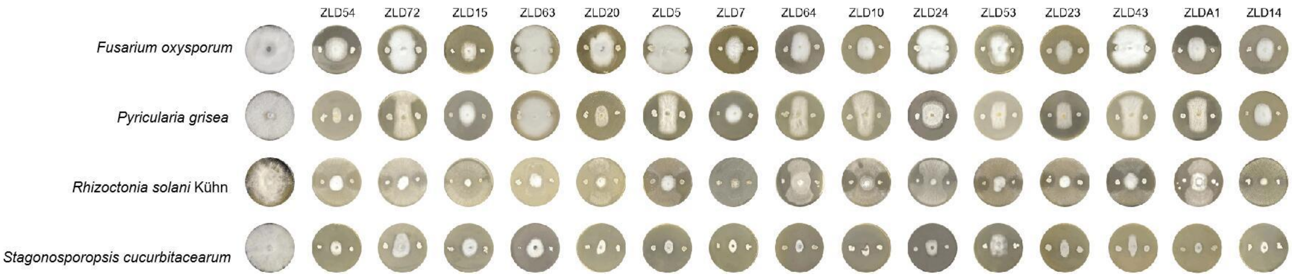

Figure S1. The broad-spectrum antifungal effects of the acnitomycete isolates.

Figure S2.

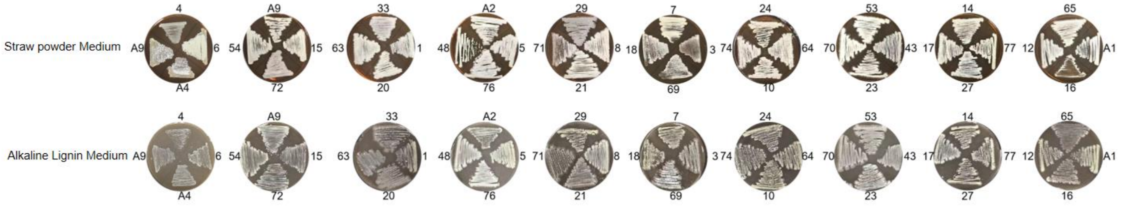

Figure S2. The growth capacity of strains on straw powder medium and alkaline lignin medium.
